# Enhanced 3-Hydroxypropionic Acid Production From Acetate *via* the Malonyl-CoA Pathway in *Corynebacterium glutamicum*

**DOI:** 10.3389/fbioe.2021.808258

**Published:** 2022-01-13

**Authors:** Zhishuai Chang, Wei Dai, Yufeng Mao, Zhenzhen Cui, Zhidan Zhang, Zhiwen Wang, Hongwu Ma, Tao Chen

**Affiliations:** ^1^ Frontier Center for Synthetic Biology and Key Laboratory of Systems Bioengineering of the Ministry of Education, School of Chemical Engineering and Technology, Department of Biochemical Engineering and Technology, Tianjin University, Tianjin, China; ^2^ Key Laboratory of Systems Microbial Biotechnology, Tianjin Institute of Industrial Biotechnology, Chinese Academy of Sciences, Tianjin, China

**Keywords:** 3-hydroxypropionic acid, acetate, *Corynebacterium glutamicum*, metabolomics analysis, fed-batch fermentation, metabolic engineering, malonyl-CoA pathway

## Abstract

Acetate is an economical and environmental-friendly alternative carbon source. Herein, the potential of harnessing *Corynebacterium glutamicum* as a host to produce 3-hydroxypropionic acid (3-HP) from acetate was explored. First, the expression level of malonyl-CoA reductase from *Chloroflexus aurantiacus* was optimized through several strategies, strain Cgz2/sod-N-C* showed an MCR enzyme activity of 63 nmol/mg/min and a 3-HP titer of 0.66 g/L in flasks. Next, the expression of citrate synthase in Cgz2/sod-N-C* was weakened to reduce the acetyl-CoA consumption in the TCA cycle, and the resulting strain Cgz12/sod-N-C* produced 2.39 g/L 3-HP from 9.32 g/L acetate. However, the subsequent deregulation of the expression of acetyl-CoA carboxylase genes in Cgz12/sod-N-C* resulted in an increased accumulation of intracellular fatty acids, instead of 3-HP. Accordingly, cerulenin was used to inhibit fatty acid synthesis in Cgz14/sod-N-C*, and its 3-HP titer was further increased to 4.26 g/L, with a yield of 0.50 g 3-HP/g-acetate. Finally, the engineered strain accumulated 17.1 g/L 3-HP in a bioreactor without cerulenin addition, representing the highest titer achieved using acetate as substrate. The results demonstrated that *Corynebacterium glutamicum* is a promising host for 3-HP production from acetate.

## Introduction

3-Hydroxypropionic acid (3-HP) is an important chemical raw material. It has been broadly used in agriculture, food, and materials (Schwarz et al., 2004), which could be attributed to its capability to produce various chemicals like 1,3-propanediol and acrylic acid (Kumar et al., 2013) and its significant market value. It is worth mentioning that 3-HP has been listed as one of the top high value-added chemicals for development both in 2004 and 2010 by the U.S. Department of Energy (Werpy et al., 2004; Bozell and Petersen, 2010). Bioconversion of 3-HP has already been extensively studied in many organisms, such as *Escherichia coli*, *Klebsiella pneumoniae*, and *Saccharomyces cerevisiae* (Rathnasingh et al., 2010; Chen et al., 2014; Zhao et al., 2019), and a variety of substrates have been exploited to produce 3-HP (Table 1). So far, the highest 3-HP titer was achieved in *Halomonas bluephagenesis*, which accumulated 154 g/L 3-HP in a 7-L bioreactor using 1,3-propanediol as substrate (Jiang et al., 2021). *Corynebacterium glutamicum* is generally recognized as safe (GRAS) with strong robustness (Richmond, 1999), which is also endowed with a broad spectrum of substrates such as xylose, glycerol, and starch (Gopinath et al., 2011). Meanwhile, it has been successfully exploited to produce various kinds of valuable chemicals, like L-glutamate, L-lysine, succinate, and acetoin with high productivities, some of which have been industrialized (Becker and Wittmann, 2012; Mao et al., 2017; Mao et al., 2018; Lu et al., 2020).

**TABLE 1:** 3-HP titers and yields of different substrates.

Organism	Carbon source	Operational technique	Titer (g/L)	Yield (g/g)	References
*Corynebacterium glutamicum* ^a^	Glucose	Fed-batch, 5-L bioreactor	62.6	0.51	Chen et al. (2017)
*Klebsiella pneumoniae* ^a^	Glycerol	Fed-batch, 5-L bioreactor	102.6	-	Zhao et al. (2019)
*Escherichia coli* ^a^	Acetate	Whole-cell biocatalysis	15.8	0.71	Lai et al. (2021)
*Corynebacterium glutamicum* ^a^	Acetate	Fed-batch, 5-L bioreactor	17.1	0.10	This study
*Halomonas bluephagenesis* ^a^	1,3-Propanediol	Fed-batch, 7-L bioreactor	154	0.93	Jiang et al. (2021)
*Escherichia coli* ^a^	Fatty acids	Fed-batch, 5-L bioreactor	52	1.56	Liu et al. (2019)

a Engineered microorganisms.

Acetic acid, a kind of non-food resource, has attracted much attention until now. It could be produced both from cheap chemical synthesis and wasted organic raw materials, which makes it a green and recyclable substrate. Although acetate is toxic and unfavorable for many microorganisms as substrate, it has been exploited to produce various kinds of organic acids such as succinate (Niu et al., 2018), itaconic acid (Noh et al., 2018), and 3-HP (Lee et al., 2018). It is worth mentioning that acetate could act as the sole carbon source in *C. glutamicum* ATCC 13032*,* when more than 6-fold of acetyl-CoA would be accumulated compared to glucose (Wendisch et al., 1997; Wendisch et al., 2000), which would be advantageous for 3-HP production through the malonyl-CoA pathway. Currently, only few research studies have focused on 3-HP production via acetate, the majority of which used *E. coli* as the host. Lee et al. (2018) modified an *E. coli* strain for 3-HP production, and it could produce 3 g/L 3-HP from 8.98 g/L acetate in shake flasks. A recombinant *E. coli* strain produced 7.3 g/L 3-HP with a yield of 0.39 g-3-HP/g-acetate in a 2.5-L bioreactor, using a two-stage strategy whereby glucose was used for cell growth and acetate for 3-HP formation (Lama et al., 2021). Recently, a whole-cell biocatalysis method was used for 3-HP production from acetate by using an engineered *E. coli* strain LNY07(M*DA), and 15.8 g/L 3-HP was produced with a yield of 0.71 g-3-HP/g-acetate (Lai et al., 2021). These studies proved the feasibility of 3-HP production from acetate.

**FIGURE 1:**
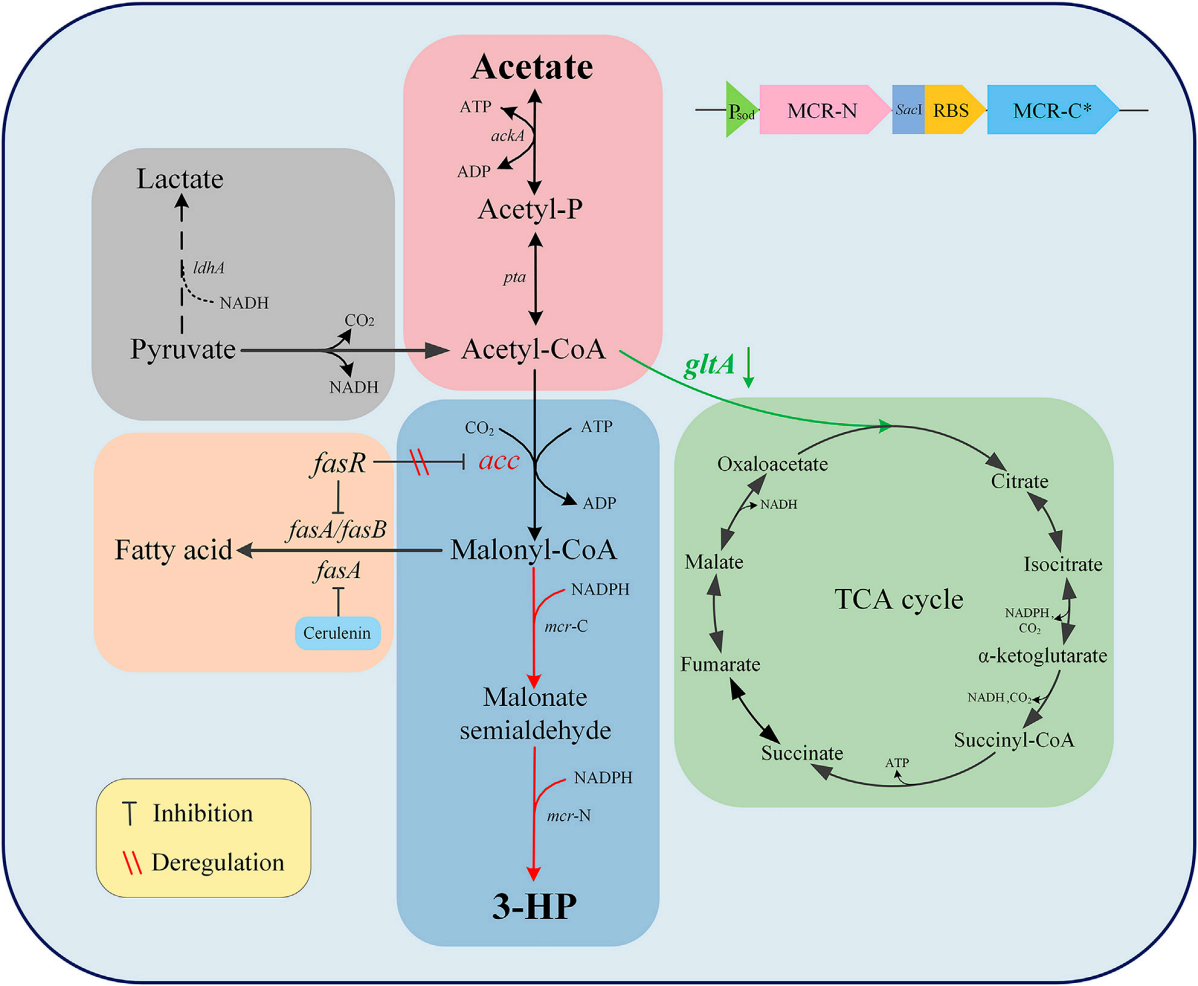
Schematic diagram of 3-HP biosynthetic route in *Corynebacterium glutamicum via* acetate assimilation. Black arrows indicate the native pathways of *C. glutamicum*, red arrows indicate genes which were overexpressed or introduced, green arrows indicate genes which were weakened, and dotted arrows indicate genes which were deleted. Acetyl-P, acetyl phosphate; 3-HP, 3-hydroxypropionic acid; *ldhA*, lactate dehydrogenase; *ackA*, acetate kinase A; *pta*, acetyl phosphate transferase; *acc*, acetyl-CoA carboxylase; *mcr-*C, C-terminal of malonyl-CoA reductase; *mcr-*N, N-terminal of malonyl-CoA reductase; *fasA*, fatty acid synthetase A; *fasB*, fatty acid synthetase B; *fasR*, fatty acid repressor; *gltA*, citrate synthase.

In our previous study, it was demonstrated that *C. glutamicum* is a promising 3-HP producer which produced 3.77 g/L 3-HP from a mixture of glucose and acetate via the malonyl-CoA pathway with cerulenin addition (Chang et al., 2020). In this study, we aimed to enhance the ability of *C. glutamicum* to efficiently produce 3-HP using acetate as the sole substrate (Figure 1). First, the expression level of malonyl-CoA reductase (MCR) was optimized to increase its activity and 3-HP accumulation in strain Cgz2. Second, gradient weakening of the expression level of citrate synthase (CS) was undertaken to save more acetyl-CoA for malonyl-CoA formation. Afterward, deregulation of acetyl-CoA carboxylase (ACC) was performed in order to increase the synthesis of malonyl-CoA. Meanwhile, metabolomics analysis was conducted to shed light on the changes of key intracellular metabolites among the engineered strains. Based on these strategies, the best strain Cgz14/sod-N-C* accumulated 4.26 g/L 3-HP with a yield of 0.50 g/g-acetate in flask cultivation with cerulenin addition. In fed-batch cultivation, the strain produced 17.1 g/L 3-HP in a 5-L bioreactor without addition of cerulenin. As far as we know, this is the highest 3-HP titer achieved by fed-batch fermentation using acetate as substrate.

## Materials and Methods

### Construction of Plasmids and *C. glutamicum* Mutant Strains

The original strain was *C. glutamicum* ATCC 13032. All the plasmids and strains used in this study are listed in Table 2. All primers used in this study are listed in Supplementary Table S1. *E. coli* DH5α was used as the host for plasmid construction. All DNA manipulations including restriction enzyme digestion and vector isolation were carried out using standard protocols. The *mcr* gene encoding MCR (NCBI-protein ID: AAS20429.1) was synthesized by GENEWIZ (Suzhou, China), and three reported point mutations (N940V, K1106W, S1114R) were introduced to increase enzyme activity (Liu et al., 2016). To construct plasmid pEC-*mcr*, gene *mcr* was amplified by PCR, followed by digesting and ligating to corresponding sites of vector pEC-XK99E. Plasmids pEC-mcr*, pEC-N-C, pEC-N-C*, and pEC-C*-N were constructed analogously. To construct plasmid pEC-mbp-mcr*, gene mcr* was amplified with a forward primer containing mbp sequence, after which the resulting fragment was digested and ligated to corresponding sites of vector pEC-XK99E. Plasmid pEC-his-mcr* was constructed analogously. To construct plasmid pEC-sod-N-C*, the promoter and RBS sequence of gene *sod* were amplified from the *C. glutamicum* ATCC 13032 genome and fused with the N-C* sequence which was amplified from pEC-N-C*. The resulting fragment and the linear fragment of pEC-XK99E without promoter P_trc_ and gene *lacIq* were digested and ligated to construct the vector. The plasmid pEC-H36-N-C* was constructed analogously. To construct plasmid pEC-sod*-mbp-N-C*, N-C* sequence was amplified with a forward primer containing mbp sequence. The resulting fragment, along with the linear fragment of pEC-sod-N-C*, was digested and ligated to construct the vector. All constructs used the RBS sequence AAAGGAGGACAACC, except for genes placed right behind P_sod_, whose RBS sequences were the same as that of gene *sod* in *C. glutamicum* ATCC 13032. Plasmids without promoter substitution need isopropyl-β-D-1-thiogalactopyranoside (IPTG) to induce expression.

**TABLE 2:** Bacterial strains and plasmids used in this study.

Strains and plasmids	Description	Source
Strains		
*Escherichia coli* DH5α	Host for plasmid construction	Invitrogen
ATCC 13032	*C. glutamicum* wild type, biotin auxotrophic	ATCC^a^
Cgz2	ATCC 13032 Δ*ldhA*	Zhu et al. (2014)
Cgz8	Cgz2 P1-*gltA*	This study
Cgz9	Cgz2 P5 *gltA*	This study
Cgz10	Cgz2 P7-*gltA*	This study
Cgz11	Cgz8 with the replacement of *gltA* initiation codon ATG by GTG	This study
Cgz12	Cgz8 with the replacement of *gltA* initiation codon ATG by TTG	This study
Cgz13	Cgz2 with mutated *fasO* sequences upstream of *accBC* and *accD1*	This study
Cgz14	Cgz12 with mutated *fasO* sequences upstream of *accBC* and *accD1*	This study
Cgz2/*mcr*	Cgz2 harboring plasmid pEC-*mcr*	This study
Cgz2/mcr*	Cgz2 harboring plasmid pEC-mcr*	This study
Cgz2/mbp-mcr*	Cgz2 harboring plasmid pEC-mbp-mcr*	This study
Cgz2/his-mcr*	Cgz2 harboring plasmid pEC-his-mcr*	This study
Cgz2/N-C	Cgz2 harboring plasmid pEC-N-C	This study
Cgz2/N-C*	Cgz2 harboring plasmid pEC-N-C*	This study
Cgz2/C*-N	Cgz2 harboring plasmid pEC-C*-N	This study
Cgz2/H36-N-C*	Cgz2 harboring plasmid pEC-H36-N-C*	This study
Cgz2/sod-N-C*	Cgz2 harboring plasmid pEC-sod-N-C*	This study
Cgz2/sod-mbp-N-C*	Cgz2 harboring plasmid pEC-sod-mbp-N-C*	This study
Cgz8/sod-N-C*	Cgz8 harboring plasmid pEC-sod-N-C*	This study
Cgz9/sod-N-C*	Cgz9 harboring plasmid pEC-sod-N-C*	This study
Cgz10/sod-N-C*	Cgz10 harboring plasmid pEC-sod-N-C*	This study
Cgz11/sod-N-C*	Cgz11 harboring plasmid pEC-sod-N-C*	This study
Cgz12/sod-N-C*	Cgz12 harboring plasmid pEC-sod-N-C*	This study
Cgz13/sod-N-C*	Cgz13 harboring plasmid pEC-sod-N-C*	This study
Cgz14/sod-N-C*	Cgz14 harboring plasmid pEC-sod-N-C*	This study
Plasmids		
pEC-XK99E	Kan^R^ ^b^, *E. coli*/*C. glutamicum* shuttle vector	Kirchner and Tauch (2003)
pEC-*mcr*	Kan^R^, pEC-XK99E containing gene *mcr* from *C. aurantiacus* (codon optimized)	This study
pEC-mcr*	Kan^R^, pEC-XK99E containing mutated gene *mcr* (N940V K1106W S1114R)	This study
pEC-mbp-mcr*	Kan^R^, pEC-mcr* with mbp tag sequence inserted between promoter and RBS	This study
pEC-his-mcr*	Kan^R^, pEC-mcr* with his tag sequence inserted between promoter and RBS	This study
pEC-N-C	Kan^R^, pEC-XK99E containing separated *mcr* gene, *mcr*-N and *mcr*-C	This study
pEC-N-C*	Kan^R^, pEC-N-C with mutated *mcr*-C	This study
pEC-C*-N	Kan^R^, pEC-N-C* with the order of *mcr*-C* and *mcr*-N exchanged	This study
pEC-H36-N-C*	Kan^R^, pEC-N-C* with P_trc_ substituted by P_H36_	This study
pEC-sod-N-C*	Kan^R^, pEC-N-C* with P_trc_ substituted by P_sod_	This study
pEC-sod-mbp-N-C*	Kan^R^, pEC-sod-N-C* with mbp tag sequence inserted between promoter and RBS	This study
pEC-mbp-N-C*	Kan^R^, pEC-N-C* with mbp tag sequence inserted between promoter and RBS	This study
pD-*sacB*	Kan^R^, vector for in-frame deletion	Zhu et al. (2013)
pD-*sacB*-P1-*gltA*	pD-*sacB* containing P_P1_ and *gltA* flanks	This study
pD-*sacB*-P5-*gltA*	pD-*sacB* containing P_P5_ and *gltA* flanks	This study
pD-*sacB*-P7-*gltA*	pD-*sacB* containing P_P7_ and *gltA* flanks	This study
pD-*sacB*-P1-GTG-*gltA*	pD-*sacB*-P1-*gltA* with translation initiation codon ATG substituted by GTG	This study
pD-*sacB*-P1-TTG-*gltA*	pD-*sacB*-P1-*gltA* with translation initiation codon ATG substituted by TTG	This study
pD-*sacB*-*fasO*(M)-*accBC*	pD-*sacB* containing the flanking sequences of *fasO* site upstream of *accBC* for mutating *fasO*	This study
pD-*sacB*-*fasO*(M)-*accD1*	pD-*sacB* containing the flanking sequences of *fasO* site upstream of *accD1* for mutating *fasO*	This study

a American Type Culture Collection.

b Kanamycin resistance.

Genome editing of *C. glutamicum* was achieved via a two-step homologous recombination using suicide plasmid pD-*sacB*. For replacing the native promoter of *gltA*, the vector pD-*sacB*-P1-*gltA* was constructed as follows: Both the upstream and downstream sequences of *gltA* promoter were amplified by PCR and fused with promoter P1, followed by digesting and ligating to corresponding sites of vector pD-*sacB*. The plasmids pD-*sacB*-P5-*gltA* and pD-*sacB*-P7-*gltA* were constructed analogously.

To introduce mutations into *fasO* sites of *accBC* and *accD1*, the vector pD-*sacB*-*fasO*(M)-*accBC* was constructed as follows: the flanking regions of the *fasO* site of *accBC* with relevant modifications were amplified and fused using PCR, after which the fused fragment was digested and ligated to corresponding sites of vector pD-*sacB*. The plasmid pD-*sacB*-*fasO*(M)-*accD1* was constructed analogously.

### Culture Conditions

For the cultivation of the plasmid host, *E. coli* DH5α was incubated at 37°C and 220 rpm in test tubes containing 5 ml LB medium; 40 μg/ml kanamycin was added to the medium if needed.

For the shake flask cultivation of various recombinant *C. glutamicum* strains, a single colony was used to inoculate 5 ml BHI broth (74 g/L) in a test tube for overnight pre-cultivation, after which 1 ml of the seed was used to inoculate 50 ml CGIII medium (10 g/L yeast extract, 10 g/L tryptone, 21 g/L MOPS, 2.5 g/L NaCl, pH 7.0) with 20 g/L glucose in a 250-ml flask. When OD_600_ reached 15–20, the culture was used to inoculate 50 ml CGXII-YA medium (10 g/L yeast extract, 14 g/L sodium acetate, 20 g/L (NH_4_)_2_SO_4_, 5 g/L urea, 1 g/L KH_2_PO_4_, 1 g/L K_2_HPO_4_, 0.25 g/L MgSO_4_·7H_2_O, 21 g/L MOPS, 10 mg/L CaCl_2_, 10 mg/L FeSO_4_·7H_2_O, 0.1 mg/L ZnSO_4_·7H_2_O, 0.2 mg/L CuSO_4_·5H_2_O, 20 μg/L NiCl_2_·H_2_O, 0.2 mg/L biotin, pH 7.0) in a 250-ml flask to an initial OD_600_ of 0.5. Then 25 μg/ml kanamycin and 1 mM isopropyl-β-D-1-thiogalactopyranoside (IPTG) at 0 h were added to the medium if needed. And 15 μM cerulenin was added to the medium at 12 h if needed. All fermentations were performed at 30°C and 220 rpm.

The seed used for fed-batch cultivation was prepared in the same way, and 200 ml seed cultured in CGIII medium was used to inoculate CGXII-YB medium (20 g/L yeast extract, 1.4 g/L sodium acetate, 20 g/L (NH_4_)_2_SO_4_, 5 g/L urea, 1 g/L KH_2_PO_4_, 1 g/L K_2_HPO_4_, 0.25 g/L MgSO_4_·7H_2_O, 10 mg/L CaCl_2_, 10 mg/L FeSO_4_·7H_2_O, 0.1 mg/L ZnSO_4_·7H_2_O, 0.2 mg/L CuSO_4_·5H_2_O, 20 μg/L NiCl_2_·H_2_O, 0.2 mg/L biotin, pH 7.0) to a working volume of 2 L in a 5-L bioreactor (Baoxing Bio, Shanghai, China). Temperature was set at 30 °C, and air flow rate was set at 1 vvm. The initial agitation speed was 300 rpm, which was set to adjust automatically to maintain dissolved oxygen above 30% of saturation. Pure acetate was added automatically into the broth to maintain pH at 7.0, and an adequate amount of sodium acetate was added externally when needed.

### Analytical Methods

Cell growth was monitored by measuring the optical density at 600 nm (OD_600_). Organic acids were quantified using a high-performance liquid chromatography (HPLC) system (Agilent Technologies, CA, United States) equipped with a cation-exchange column (HPX-87H; BioRad, CA, United States), as described previously (Mao et al., 2017; Mao et al., 2018). And 5 mM H_2_SO_4_ was used as mobile phase at a flow rate of 0.4 ml/min, and temperature of the column was set at 65°C. All data represent the average values and standard deviations from three independent replicates.

### Enzyme Activity Assays

To extract crude enzyme, *C. glutamicum* strains were cultured in CGXII-YA medium till the exponential growth phase. Procedures of crude enzyme extraction were followed as previously described (Lu et al., 2020). Total protein concentrations were determined using a Bradford assay kit (CWBiotech, Beijing, China).

For measuring the enzyme activity of MCR, the procedures were followed as previously described (Liu et al., 2013) with incubation temperature changed to 30 °C. The enzyme activity of MCR was calculated according to the oxidation rate of NADPH.

For measuring the enzyme activity of CS, the procedures were followed as previous described (Eikmanns et al., 1994). The enzyme activity of CS was calculated according to the formation rate of coenzyme A.

### Metabolome Analysis

Procedures for extraction of intracellular metabolites are listed in the Supplementary Material. Intracellular metabolites were analyzed using an ultra-performance liquid chromatography (UPLC) system (Nexera 30A, Shimadzu, Kyoto, Japan) coupled with a mass spectrometer (TripleTOF^™^ 5600, Applied Biosystem Sciex, United States) in negative electrospray ionization (ESI) mode. Most of the metabolites were identified with LC equipped with a SeQuant ZIC-HILIC column (100 × 2.1 mm, 3.5 μm, Merck, Germany). Then 10 mM ammonium acetate and 100% acetonitrile were used as mobile phases A and B, respectively, and the flow rate was set at 0.2 ml/min with a gradient as follows: 0–3 min, 90% B; 3–6 min, 90–60% B; 6–25 min, 60–50% B; 25–30 min, 50% B; 30–30.5 min, 50–90% B; and 30.5–38 min, 90% B. The relative content of metabolites was normalized to cell density.

### Real-Time Quantitative PCR

To extract RNA, *C. glutamicum* strains were cultured in CGXII-YA medium till the exponential growth phase. Procedures of total RNA extraction and qRT-PCR were followed as previously described (Zhu et al., 2013). The transcriptional level of 16S ribosomal RNA of *C. glutamicum* was used as an internal reference. Each targeted gene of a strain was measured three times.

## Results and Discussion

### Optimization of the Expression Level of Malonyl-CoA Reductase

**FIGURE 2:**
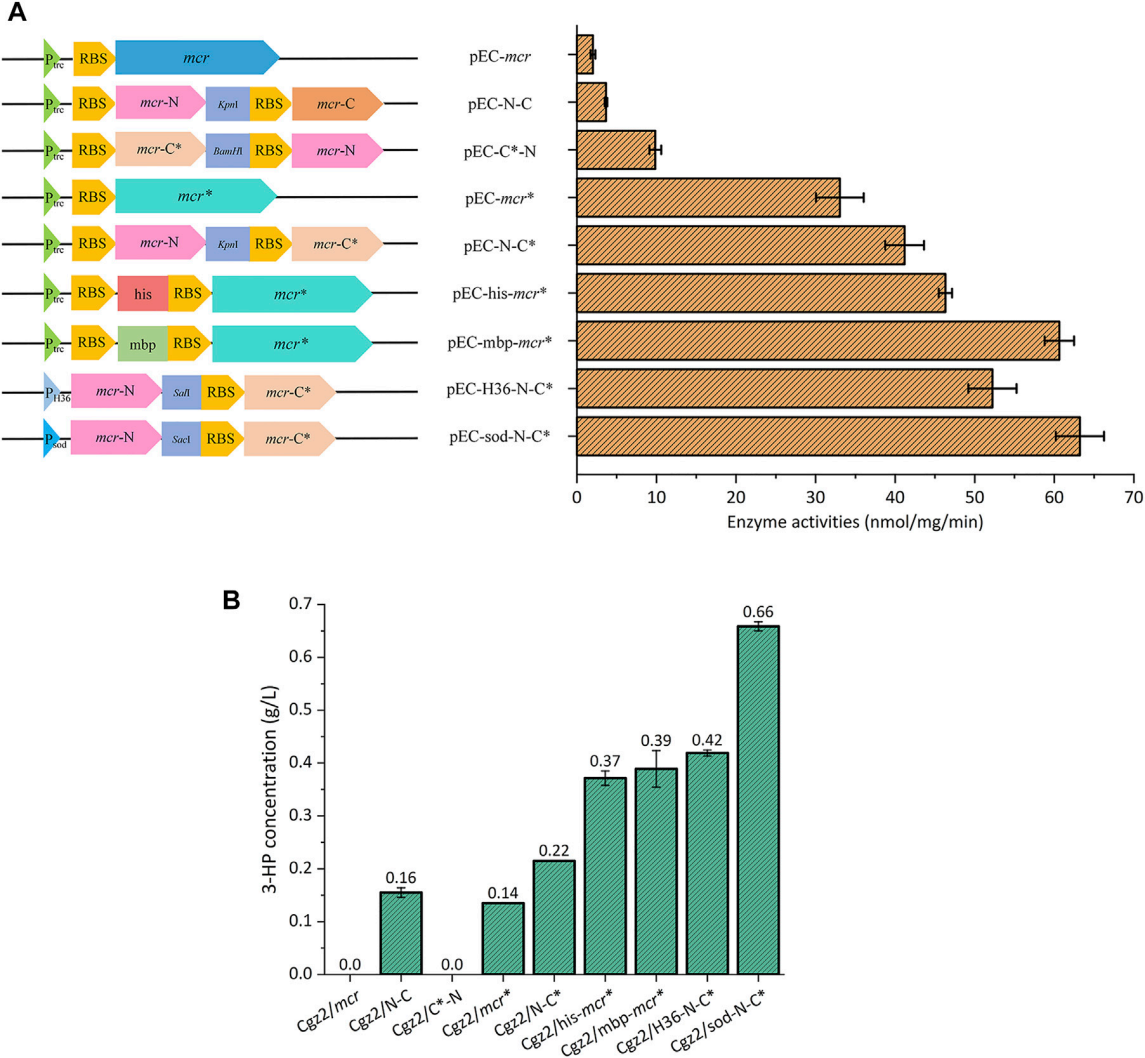
Overviews of a series of *mcr* overexpression plasmids and their applications in 3-HP production. **(A)** Schematic drawing of different *mcr* overexpression plasmids, and their respective MCR enzyme activities. **(B)** 3-HP concentrations of Cgz2 series strains.

*Corynebacterium glutamicum* can assimilate acetate as the sole carbon substrate. More precursor acetyl-CoA would be supplemented when acetate was utilized, rather than glucose, which could be beneficial to 3-HP production via malonyl-CoA pathway. However, *C. glutamicum* is not a natural 3-hydroxypropionic acid (3-HP) producer due to the lack of the malonyl-CoA reductase (MCR). Therefore, the mcr* gene encoding a bifunctional malonyl-CoA reductase mutant (N940V, K1106W, S1114R) (Liu et al., 2016) from *Chloroflexus aurantiacus* was inserted into shuttle vector pEC-XK99E, resulting in pEC-mcr*. Then, several strategies were used to increase the expression level of MCR (Figure 2A): the sequences of mbp-tag and his-tag, which could increase the expression level of target protein by destabilizing the secondary structures of mRNA (Fang et al., 2018), were inserted upstream of the RBS of mcr*, resulting in expression plasmids pEC-mbp-mcr* and pEC-his-mcr*, respectively; the N-terminal half with malonate semialdehyde (MSA) reductase (MCR-N) activity and the C-terminal half with malonyl-CoA reductase (MCR-C) activity (Liu et al., 2013) were separately expressed in different sort orders, yielding plasmids pEC-N-C, pEC-N-C*, and pEC-C*-N; and stronger promoters P_H36_ (Yim et al., 2013) and P_sod_ were used to express N-terminal and C-terminal halves, resulting in pEC-H36-N-C* and pEC-sod-N-C*, respectively. The vector pEC-*mcr* which contains unmutated *mcr* gene was also constructed as control. All plasmids were separately introduced into Cgz2, generating a series of strains (Table 2). An optimization of IPTG induction strength was conducted, and an induction time and concentration of 0 h and 1 mM were found to be ideal for MCR expression (Supplementary Figure S1). Both MCR enzyme activities and 3-HP titers of the constructed strains were determined.

As is shown in Figure 2A, Cgz2/*mcr* showed neglectable MCR activity (2 nmol/mg/min), which explained the undetectable 3-HP in the medium. MCR activity of Cgz2/mcr* was increased to 33 nmol/mg/min, which was 15.5-fold higher than Cgz2/*mcr*, and 0.14 g/L 3-HP (Figure 2B) was accumulated by this strain in 48 h. When mbp-tag and his-tag were introduced, the corresponding strains Cgz2/mbp-mcr* and Cgz2/his-mcr* both showed an obvious increase in MCR enzyme activity and 3-HP titer. The better producer Cgz2/mbp-mcr* accumulated 0.42 g/L 3-HP. Strain Cgz2/N-C* possessing both mutation and dissection showed higher catalytic efficiency (41 nmol/mg/min), and 3-HP titer was also increased to 0.22 g/L. It was reported that C-terminal half was the rate-limiting part (Liu et al., 2016); therefore, it is reasonable to deduce that placing *mcr-*C* into the first place of the operon would improve its expression level as a similar case was reported (Liu et al., 2019). However, an obvious decline of catalytic activity (9.9 nmol/mg/min) in Cgz2/C*-N was observed, resulting in the vanishment of 3-HP (Figure 2B). Both strains Cgz2/sod-N-C* (63 nmol/mg/min) and Cgz2/H36-N-C* (52 nmol/mg/min) showed much increased MCR enzyme activity. The best strain Cgz2/sod-N-C* produced 0.66 g/L 3-HP after 48 h cultivation in flasks (Supplementary Figure S2). Cell growth was not severely retarded when acetate was used as the sole carbon source, and the biomass of Cgz2/sod-N-C* reached 23.8 OD_600_. The results illustrated the successful optimization of MCR expression in *C. glutamicum*.

The mbp-tag and his-tag were shown to improve the enzyme activity of MCR and the overall 3-HP production under the control of promoter P_trc_. The mRNA secondary structure analysis showed that the insertion of mbp-tag and his-tag increased the minimum free energy (MFE) from -36.15 kJ/mol to -27.24 kJ/mol and -24.8 kJ/mol, respectively (Supplementary Figure S3), which connoted the effectiveness of mbp-tag and his-tag in destabilizing the mRNA secondary structure. Moreover, transcriptional levels of mcr* were also measured by RT-PCR. As shown in Supplementary Figure S4, mRNA levels of gene mcr* in Cgz2/his-mcr* and Cgz2/mbp-mcr* were slightly increased, which were 13.0 and 12.4% higher than that of Cgz2/mcr*, respectively. However, the enzyme activities of MCR in Cgz2/his-mcr* and Cgz2/mbp-mcr* were, respectively, 40.2 and 83.4% higher than the activity of Cgz2/mcr*. The results demonstrated that the increased enzyme activities of MCR in Cgz2/his-mcr* and Cgz2/mbp-mcr* were mainly caused by destabilization of mRNA secondary structure of mcr*, which facilitated the translation initiation of MCR. Naturally, the better mbp-tag was also inserted in pEC-sod-N-C* and then introduced into strain Cgz2, resulting in strain Cgz2/sod-mbp-N-C*. Minimum free energy of pEC-sod-mbp-N-C* also increased from -30.2 kJ/mol to -25.25 kJ/mol compared to pEC-sod-N-C* (Supplementary Figure S5); however, 3-HP production was not boosted as expected (Supplementary Figure S6). It was presumed that insertion of tag could only enhance the expression level of *mcr-*N, leaving the rate-limiting *mcr-*C* unaffected, which was of no help to 3-HP production. Strain Cgz2/mbp-N-C* under the control of promoter P_trc_ was also constructed to vindicate our presumption. As expected, 3-HP titer of Cgz2/mbp-N-C* did not increase comparing with strain Cgz2/N-C* (data not shown). Therefore, Cgz2/sod-N-C* was selected for further modification.

### Knockdown of Gene *gltA* Boosts the Production of 3-HP

Acetyl-CoA is the indirect key precursor for 3-HP synthesis, whose availability would extensively affect malonyl-CoA pool and 3-HP production. It was reported that when glucose was replaced with acetate as the sole carbon source, acetyl-CoA concentration in *C. glutamicum* was increased by about 5-fold, and the majority of which was depleted through the TCA cycle (Wendisch et al., 1997; Wendisch et al., 2000). In order to save more acetyl-CoA for 3-HP synthesis, we tried to reduce the flux of TCA by downregulating the expression of the *gltA* gene, which encodes citrate synthase (CS).

**FIGURE 3:**
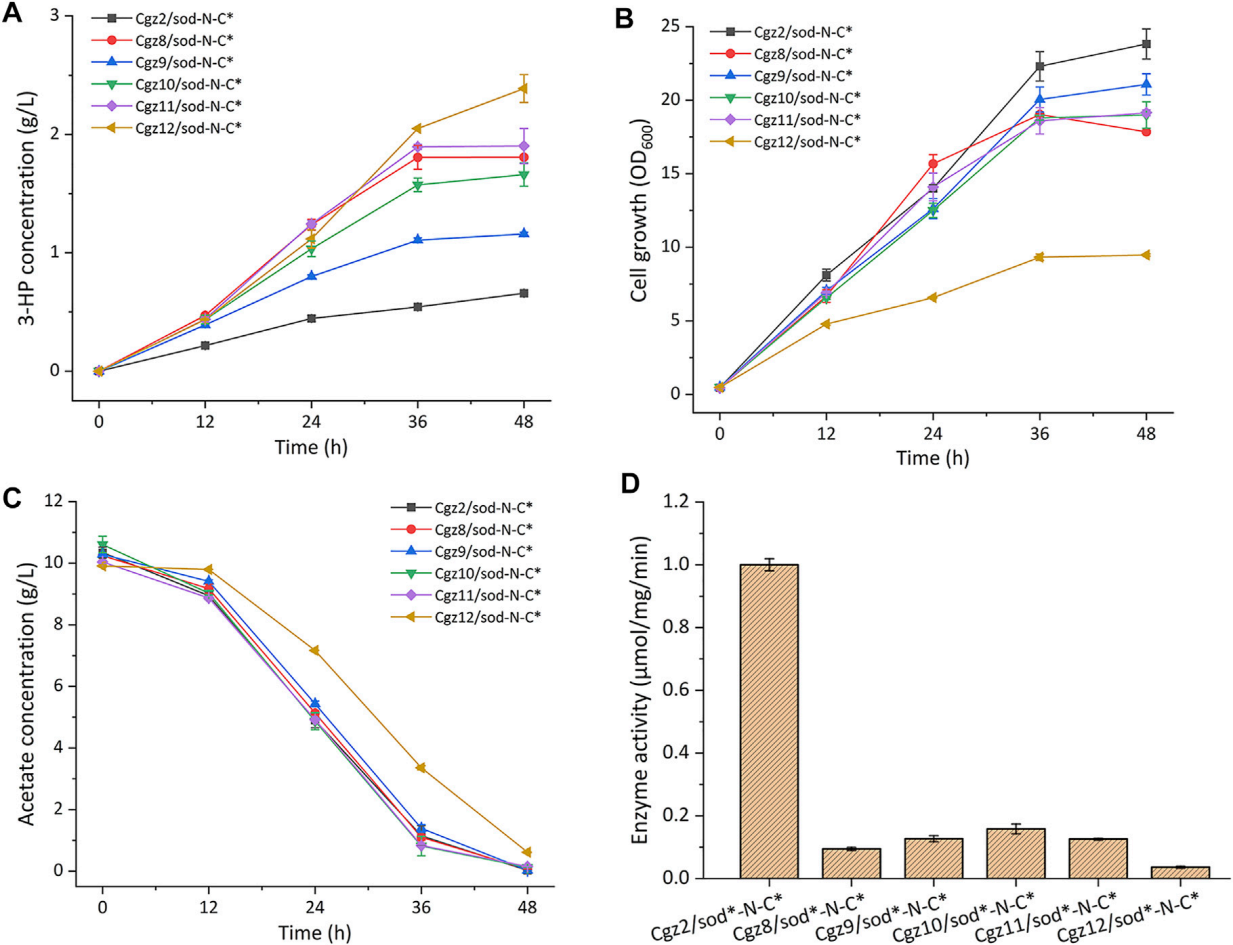
Characterizations of citrate synthase (CS) modified recombinant strains. **(A)** 3-HP production, **(B)** cell growth (OD_600_), **(C)** acetate consumption, and **(D)** CS enzyme activities.

Three weak promoters P_1_, P_5_, and P_7_ with different strengths (1, 6, and 13% relative strength of promoter P_trc_, respectively) were selected from a promoter library constructed in our previous work (Zhang et al., 2018) and used to replace the native promoter of *gltA*, respectively. Then plasmid pEC-sod-N-C* was introduced into the engineered strains, yielding strains Cgz8/sod-N-C*, Cgz9/sod-N-C*, and Cgz10/sod-N-C*. The cultivation results indicated that all the three strains exhibited significantly increased 3-HP titer, and the best producer Cgz8/sod-N-C* accumulated 1.81 g/L 3-HP, which was 1.74-fold higher than that of the control strain Cgz2/sod-N-C*, while strains Cgz9/sod-N-C* and Cgz10/sod-N-C* produced 1.16 g/L and 1.66 g/L 3-HP, respectively (Figure 3A). Meanwhile, the maximum biomass of all the three strains decreased to different extents, among which Cgz8/pEC-sod-N-C* (OD_600_ 17.85) exhibited the highest biomass reduction (25.1%) compared with Cgz2/sod-N-C* (Figure 3B). No difference in acetate consumption was observed among the strains, suggesting the acetate transport and utilization systems were not affected (Figure 3C).

Since P_P1_ is the weakest promoter in the library (Zhang et al., 2018), the start codon ATG of *gltA* was replaced with GTG and TTG, respectively, to further decrease the expression level of CS in Cgz8/sod-N-C*, generating strains Cgz11/sod-N-C* and Cgz12/sod-N-C*. As expected, cell growth of Cgz11/sod-N-C* slightly declined to 16.65 OD_600_, and 1.90 g/L 3-HP was detected, which was similar to Cgz8/sod-N-C* (Figure 3A). The 3-HP titer of Cgz12/sod-N-C* showed a more distinct increase of 32.04%, reaching 2.39 g/L with a yield of 0.26 g-3-HP/g-acetate in 48 h (Figure 3A), which were, respectively, 2.62-fold and 3.06-fold higher than those of strain Cgz2/sod-N-C*. Accordingly, the maximal biomass of Cgz12/sod-N-C* was further decreased to an OD_600_ of 9.48, which decreased by 60% compared to Cgz2/sod-N-C* (Figure 3B). The acetate consumption rate was also negatively affected by the decreased cell growth, and 0.61 g/L residual acetate was detected after the fermentation (Figure 3C).

In order to prove that it was the reduction of CS enzyme activity that escalated 3-HP production, CS enzyme activities of relevant strains were analyzed. CS activities were sharply decreased by 90.6, 84.2, and 87.3%, respectively, when promoters P_1_, P_5_, and P_7_ were used. The detected CS activities were in line with the trends of 3-HP production and cell growth (Figure 3D). Thereinto, CS enzyme activity of the best producer Cgz12/sod-N-C* with the start codon TTG for *gltA* was reduced to merely 3.62% of the control. The results demonstrated that the reduced CS activity contributed significantly to 3-HP accumulation.

### Deregulation of the Expression of *acc* Genes Elevated 3-HP Production

Malonyl-CoA is the direct precursor for 3-HP synthesis and is converted from acetyl-CoA. This reaction was catalyzed by acetyl-CoA carboxylase (ACC)—a heterodimer comprising subunits AccBC and AccD1 (Gande et al., 2007). It was reported that the two subunits were strictly regulated by transcriptional repressor FasR via binding with the *fasO* motifs of *accBC* and *accD1* (Nickel et al., 2010). Since FasR is also a negative regulator for the genes involving fatty acid synthesis pathway, which is the dominating competitive pathway of 3-HP synthesis, we chose to deregulate the expression of *acc* genes by eliminating the *fasO* sequences, instead of deleting *fasR*.

**FIGURE 4:**
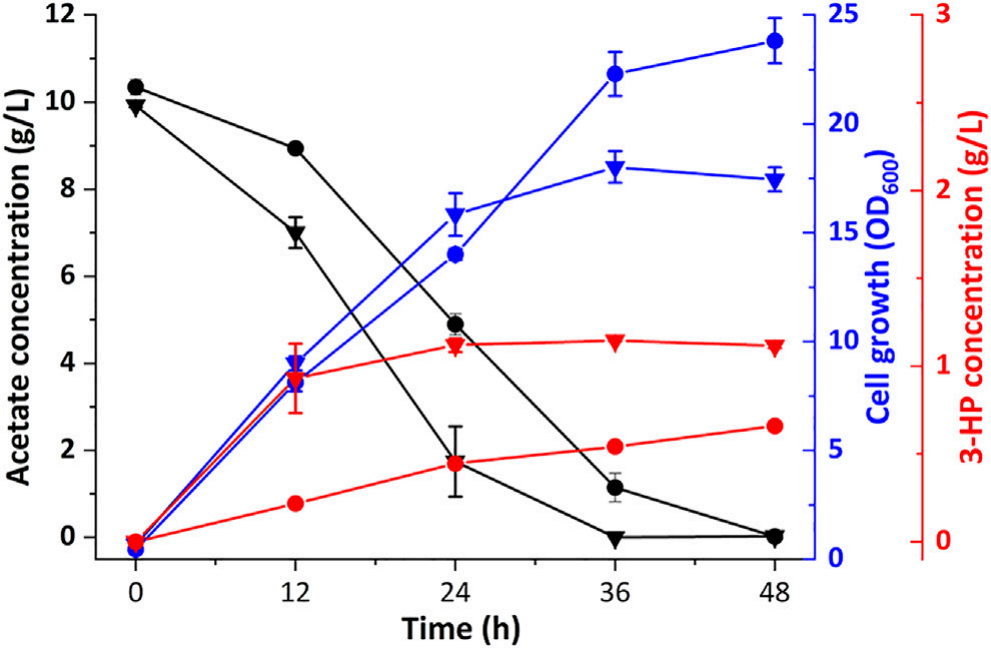
Effect delineations of deregulation of *acc*. Time profiles of cell growth (OD_600_), acetate, and 3-HP concentrations of recombinant strains Cgz2/sod-N-C* and Cgz13/sod-N-C*. Circles indicate strain Cgz2/sod-N-C*. Triangles indicate strain Cgz13/sod-N-C*.

The *fasO* sequences of *accBC* and *accD1* in Cgz2/sod-N-C* were substituted, as previously reported (Milke et al., 2019), generating strain Cgz13/sod-N-C*. As shown in Figure 4, strain Cgz13/sod-N-C* produced 1.12 g/L 3-HP in 24 h and the titer remained about the same (1.19 g/L) in 36 h as a result of the depletion of acetate in the medium, which was 1.80 times as Cgz2/sod-N-C*. Meanwhile, 3-HP yield also increased to 0.12 g-3-HP/g-acetate. Noteworthily, 3-HP productivity was increased from 18.54 mg/L/h to 46.64 mg/L/h during 0–24 h. Likewise, the acetate assimilation rate of Cgz13/sod-N-C* reached 0.34 g/L/h, which was increased by 47.8% compared with strain Cgz2/sod-N-C* (0.23 g/L/h). The cell growth rates of the two strains were similar within 24 h. However, the final OD_600_ of strain Cgz13/sod-N-C* in 36 h was only 18.0, 24% lower than that of strain Cgz2/sod-N-C*. It was concluded that deregulation of the repression of the *acc* genes could efficiently redirect more acetyl-CoA toward 3-HP production.

### Combination of the Strategies of *gltA* Knockdown and Deregulation of the Expression of *acc* Genes

Both the knockdown of *gltA* and deregulation of the expression of *acc* genes were effective in promoting 3-HP production. Hence, mutations of *acc* genes were introduced into strain Cgz12/sod-N-C*, generating strain Cgz14/sod-N-C*. However, no obvious differences in final 3-HP titer, cell growth, and acetate consumption rate were observed between the two strains (Supplementary Figure S7). The transcription levels of *accBC* and *accD1* were then analyzed by RT-PCR to verify the effect of *acc* genes deregulation. As shown in Supplementary Figure S8, the transcription levels of genes *accBC* and *accD1* in strain Cgz14/sod-N-C* were, respectively, 2.7 times and 3.0 times as those of strain Cgz12/sod-N-C*, illustrating the fact that the two *acc* genes were deregulated by *fasO* mutations.

The combination of the two strategies did not promote 3-HP production further as expected. Considering the enzyme activities of MCR were similar in Cgz2/sod-N-C* and Cgz12/sod-N-C*, it could be deduced that supply of acetyl-CoA coordinated well with the MCR activity in Cgz2/sod-N-C*. However, when CS was weakened and acetyl-CoA consumption in the TCA cycle was decreased in strain Cgz12/sod-N-C*, its MCR activity or NADPH supply became the rate-limiting factor for 3-HP production. Future endeavors should focus on further optimization of MCR expression and increase the supply of cofactor NADPH.

### Quantification of the Intracellular Metabolites *via* Metabolomics Analysis

**FIGURE 5:**
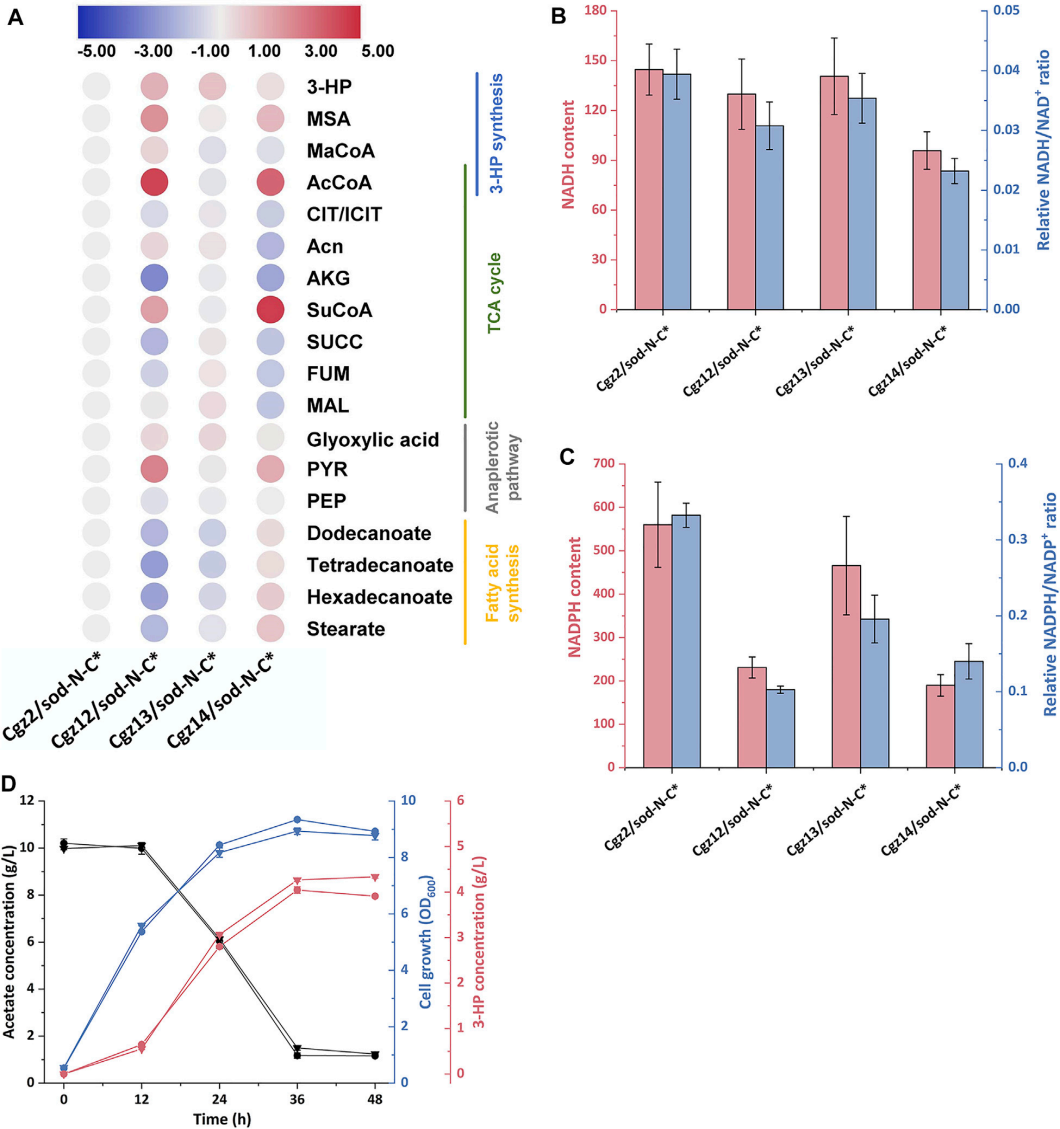
Changes of intracellular metabolites in response to different genetic manipulations. **(A)** Heat map (log_2_ fold change) showing the changes of intracellular metabolites involving central carbon metabolism in strains Cgz2/sod-N-C*, Cgz12/sod-N-C*, Cgz13/sod-N-C*, and Cgz14/sod-N-C* at 12 h. “#” indicates metabolites that could not be distinguished in LC-MS. Comparison of intracellular **(B)** relative NADH content and NADH/NAD^+^ ratio, and **(C)** relative NADPH content and NADPH/NADP^+^ ratio in strains Cgz2/sod-N-C*, Cgz12/sod-N-C*, Cgz13/sod-N-C*, and Cgz14/sod-N-C* at 12 h. **(D)** Time profile of cell growth (OD_600_), acetate, and 3-HP concentrations of Cgz12/sod-N-C* and Cgz14/sod-N-C* with addition of 15 μM cerulenin at 12 h.

To elucidate the changes of intracellular metabolites behind the improvement of 3-HP titers and give instructions to future strain improvement, intracellular metabolites of strains Cgz2/sod-N-C*, Cgz12/sod-N-C*, Cgz13/sod-N-C*, and Cgz14/sod-N-C* were extracted and analyzed. When comparing Cgz12/sod-N-C* with Cgz2/sod-N-C*, it was shown that attenuating CS had a significant influence on metabolites involving several pathways (Figure 5A). Acetyl-CoA, the main substrate for CS, was increased by 18-fold in Cgz12/sod-N-C* and consequently increased concentrations of metabolites involving 3-HP synthesis (53, 411 and 181% higher for malonyl-CoA, MSA and 3-HP, respectively). This result demonstrated that the knockdown of *gltA* directed more acetyl-CoA into the 3-HP synthesis pathway in Cgz12/sod-N-C*. However, the significantly piled up acetyl-CoA and MSA suggested the activities of ACC and MCR-N might be the limiting steps for 3-HP production. On the other hand, metabolites in the major competing route, TCA cycle, were decreased in different degrees (30, 84, 62, and 39% lower for citrate/isocitrate, α-ketoglutarate, succinate, and fumarate, respectively). Moreover, intracellular levels of cofactors NADH and NADPH were also influenced by the decreased flux through the TCA cycle as the intracellular NADH content and NADH/NAD^+^ ratio were decreased by 10 and 22%, respectively; meanwhile, the NADPH content and NADPH/NADP^+^ ratio were decreased by 59 and 69%, respectively (Figures 5B,C). The reduced concentrations of these metabolites were highly correlated with the reduced maximal cell biomass of Cgz12/sod-N-C*.

The detailed intracellular metabolites in Cgz14/sod-N-C* were also compared with Cgz12/sod-N-C* to analyze the metabolic response of deregulating *acc* genes. As shown in Figure 5A, the concentrations of metabolites along the 3-HP synthesis pathway obviously declined (44, 50, 51, and 54% lower for acetyl-CoA, malonyl-CoA, MSA, and 3-HP, respectively). On the contrary, the concentrations of the main fatty acids were much higher in Cgz14/sod-N-C* (270, 463, 560, and 414% higher for dodecanoate, tetradecanoate, hexadecanoate, and stearate, respectively). This result indicated more acetyl-CoA was directed into fatty acids, instead of 3-HP synthesis pathway in Cgz14/sod-N-C*. However, the phenomenon was not observed in Cgz13/sod-N-C* when compared with its control strain Cgz2/sod-N-C* (Figure 5A). Deregulating *acc* genes in two different hosts (Cgz2/sod-N-C* and Cgz12/sod-N-C*) led to different metabolic responses, and the exact reason for which was undetermined. It was reported that the *K*_*m*_ value for NADPH of MCR from *Chloroflexus aurantiacus* was 25 μM (Hugler et al., 2002), which was much higher than the corresponding value (4.5 μM) of fatty acid synthase from *C. glutamicum* (Agira et al., 1984)*.* Therefore, it was deduced that the difference might be caused by the different NADPH and NADH levels (or NADPH/NADP^+^ and NADH/NAD^+^ ratios) in the two hosts, and malonyl-CoA might tend to be metabolized by fatty acid synthesis pathway under a lower intracellular NADPH level (Figure 5C). Moreover, concentrations of metabolites in TCA were decreased to different extents in Cgz14/sod-N-C* (19, 75, 17, and 54% lower for citrate/isocitrate, aconitate, fumarate, and malate, respectively), which showed that the deregulation of the expression of *acc* genes further reduced the amount of acetyl-CoA metabolized in TCA cycle. The NADPH level in Cgz14/sod-N-C* was further decreased by 18%, indicating the NADPH availability in Cgz14/sod-N-C* needs to be improved to increase 3-HP synthesis.

In view of the fact that the increased supply of malonyl-CoA in Cgz14/sod-N-C* was mainly directed to fatty acids synthesis, cerulenin was used to inhibit fatty acid synthesis to explore its potential in 3-HP production with Cgz12/sod-N-C* as control. As shown in Figure 5D, the 3-HP titer and yield of Cgz14/sod-N-C* reached 4.26 g/L and 0.50 g-3-HP/g-acetate, which were 5.4 and 12.2% higher than strain Cgz12/sod-N-C*. The addition of cerulenin resulted in an increase of 81 and 108% in 3-HP titer and yield compared to those without cerulenin addition, and 3-HP productivity was also significantly increased from 50 mg/L/h to 120 mg/L/h during 0–36 h. The results demonstrated that Cgz14/sod-N-C* is a promising host for 3-HP production from acetate. However, the high cost of cerulenin renders its application unsuitable for large-scale production. Therefore, besides further increasing MCR activity, future endeavors should also be made to inhibit fatty acid synthesis properly, which can save both malonyl-CoA and NADPH for 3-HP production.

### Fed-Batch Fermentation in a 5 L Bioreactor

**FIGURE 6:**
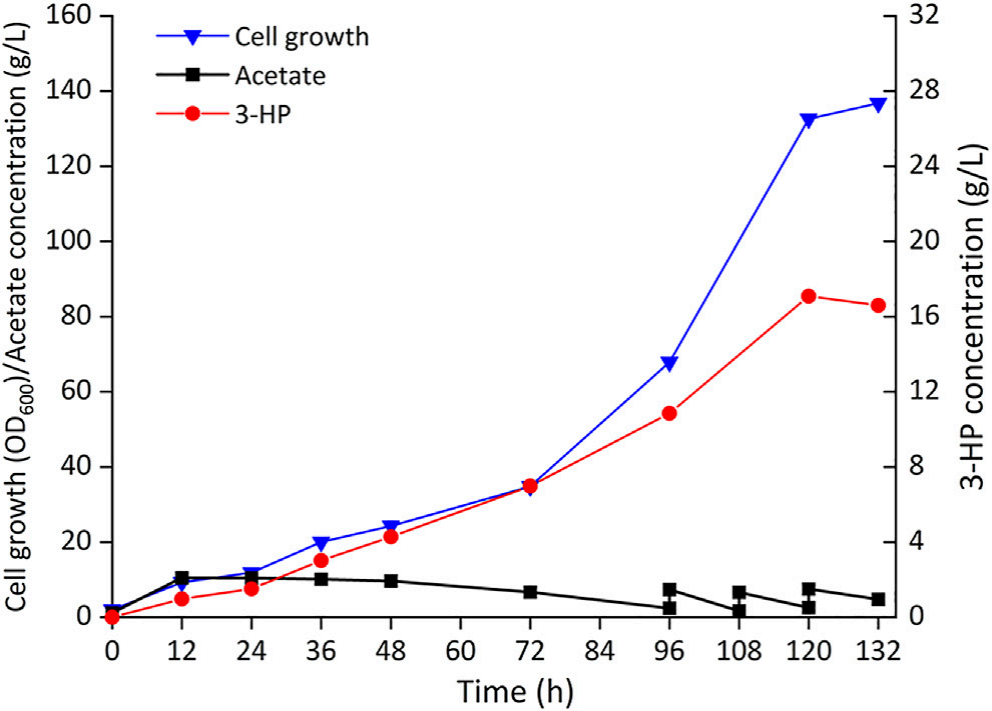
Time profiles of cell growth (OD_600_), acetate, and 3-HP concentrations during the fed-batch culture of recombinant strain Cgz14/sod-N-C* in a 5-L bioreactor. Pure acetate was added automatically into the broth to maintain pH at 7.0; 14 g sodium acetate was added externally at 96, 108, and 120 h.

To further evaluate the ability of 3-HP production from acetate, Cgz14/sod-N-C* was cultured in a 5-L bioreactor without cerulenin addition. As shown in Figure 6, a titer of 17.1 g/L 3-HP was obtained in 120 h with a production rate of 140 mg/L/h and a yield of 0.10 g-3-HP/g-acetate. Cell growth was slow in the first 72 h, after which cells grew faster with a specific growth rate of 0.028 h^−1^ till 120 h, reaching an OD_600_ of 132.6. The yield of 3-HP in bioreactor decreased by 61.5% compared with that of the same strain in shake flasks, which might be caused by increased carbon flow toward cell growth in the bioreactor, indicating the conditions for fed-batch fermentation could be further optimized.

Strain Cgz14/sod-N-C* produced 4.26 g/L 3-HP from 8.52 g/L acetate with cerulenin addition in shake flasks, and 17.1 g/L 3-HP using acetate as substrate without addition of cerulenin in bioreactor, which is the highest titer achieved using acetate as substrate. The performance of the engineered *C. glutamicum* strain proved its potential in 3-HP production from acetate.

## Conclusion

This is the first report on engineering *C. glutamicum* to sufficiently assimilate acetate as the sole substrate to produce 3-HP. With optimization of MCR expression and weakening of CS expression, 3-HP titer of strain Cgz12/sod-N-C* was increased to 2.39 g/L. With the subsequent deregulation of ACC and addition of cerulenin, strain Cgz14/sod-N-C* produced 4.26 g/L 3-HP with a yield of 0.50 g/g-acetate. Furthermore, Cgz14/sod-N-C* accumulated 17.1 g/L 3-HP without addition of cerulenin in a 5 L bioreactor. The results demonstrate that *C. glutamicum* is a promising host for 3-HP production from acetate.

## Data Availability

The original contributions presented in the study are included in the article/Supplementary Material; further inquiries can be directed to the corresponding authors.

## Abbreviations

MCR, malonyl-CoA reductase; TCA, tricarboxylic, acid; CS, citrate synthase; ACC, acetyl-CoA carboxylase.
