# Supplementary material for: Enhanced 3-Hydroxypropionic Acid Production From Acetate via the Malonyl-CoA Pathway in Corynebacterium glutamicum
Source: Front Bioeng Biotechnol. 2022 Jan 13;9:808258. doi: 10.3389/fbioe.2021.808258 (PMC8790568; doi:10.3389/fbioe.2021.808258)
Supplement: Supplementary file 1 [file DataSheet1.docx]

Supplementary Material

# Supplementary Data

## Materials

Primers were synthesized by GENEWIZ (Suzhou, China). Plasmids were extracted using Axyprep^™^ Plasmid Miniprep Kit (Axygen, New York, NY, USA), DNA fragments were purified using SanPrep Column Plasmid Mini-Preps Kit (Sangon Biotech, Shanghai, China). 3-HP standard (30% in water) was purchase from Tokyo Chemical Industry (Tokyo, Japan). BHI broth was purchased from Hopebio (Qingdao, China). Yeast extract and tryptone were purchased from OXOID (Hants, UK). MOPS (3-morpholinopropanesulfonic acid) was purchased from Solomen (Tianjin,China). Pure acetate was purchased from Damao (Tianjin, China). Other reagents were purchased from Sangon Biotech (Shanghai, China).

## Extraction of intracellular metabolites

To extract intracellular metabolites, *C. glutamicum* strains were cultured in CGXII-YA medium till exponential growth phase. 3 mL of the culture was immediately mixed with 15 mL of -20 °C 40% methanol by vortex oscillation, followed by centrifugation at 4 °C, 14,000×g (same centrifugation condition below) for 2 min. After removal of supernatant, quenched cells were resuspended by 2 mL -20 °C methanol and centrifuged for 2 min, supernatant (sample 1) was kept at -20 °C. The pellets were resuspended by 2 mL -20 °C acidic acetonitrile/water (1:1, v/v, with 0.1% formic acid) followed by 10 s oscillation and 15 min ice-water bath, during which mixtures were oscillated for 5 s every 5 min. Then mixtures were centrifuged for 5 min, supernatant (sample 2) was kept at -20 °C. The pellets were resuspended, oscillated, bathed and centrifuged again. Supernatant was added into sample 2, and pellets were resuspended by 2 mL 100 °C ethanol/water (3:1, v/v), followed by 10 s oscillation and 10 min 100 °C water bath, during which mixtures were oscillated for 5 s every 5 min. Then mixtures were centrifuged for 5 min, the supernatant was mixed with sample 1, 2 and was centrifuged for 5 min, the resultant supernatant was freeze dried and stored at -80 °C.

# Supplementary Figures and Tables

## Supplementary Figures


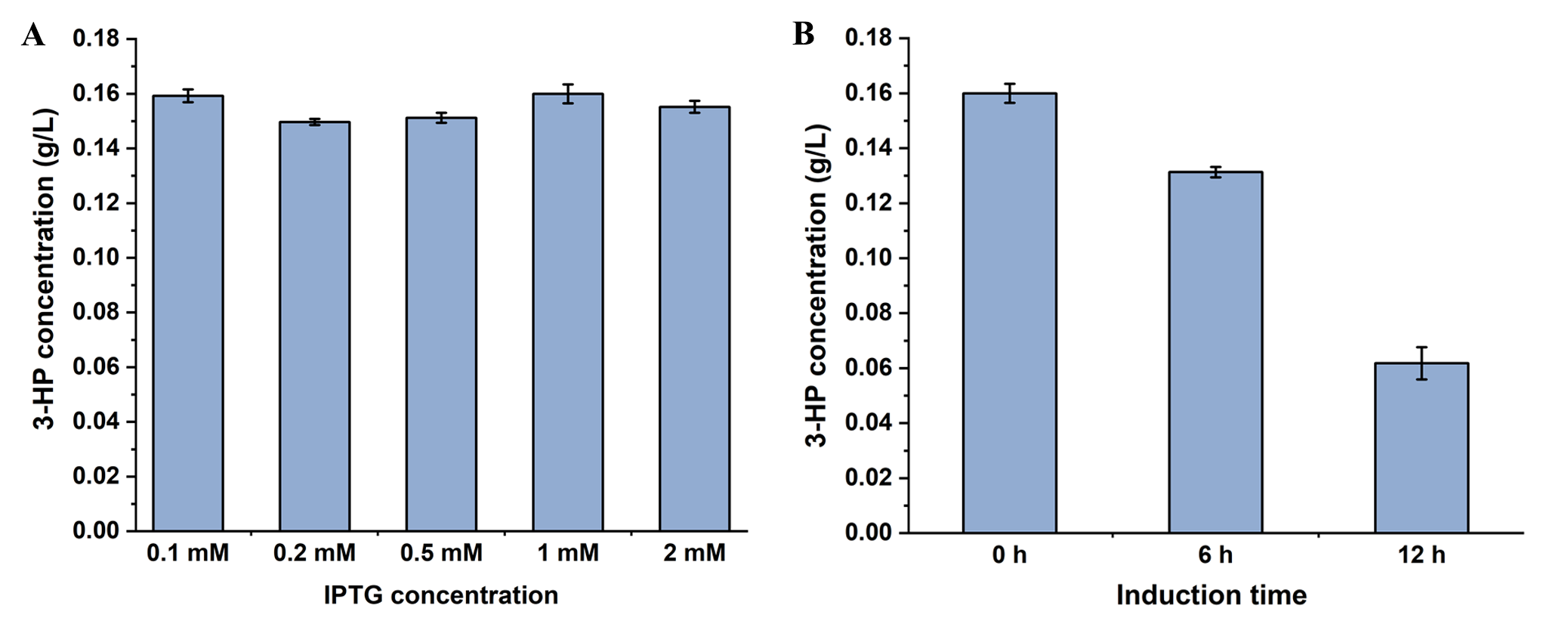


**Supplementary Figure S1.** Effect of IPTG induction strength on 3-HP production by Cgz2/*mcr**. 3-HP titer of **(A)** different IPTG concentration added into the medium at 0 h and **(B)** 1 mM IPTG added into the medium at different induction time.


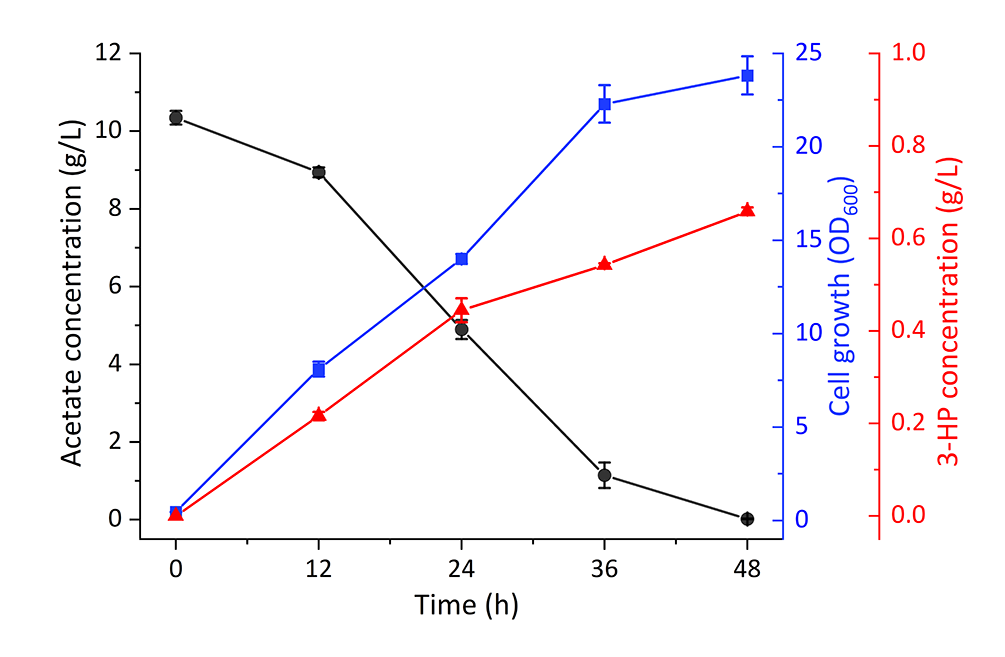


**Supplementary Figure S2.** Time profiles of cell growth (OD_600_), acetate and 3-HP concentrations of strain Cgz2/sod-N-C*.


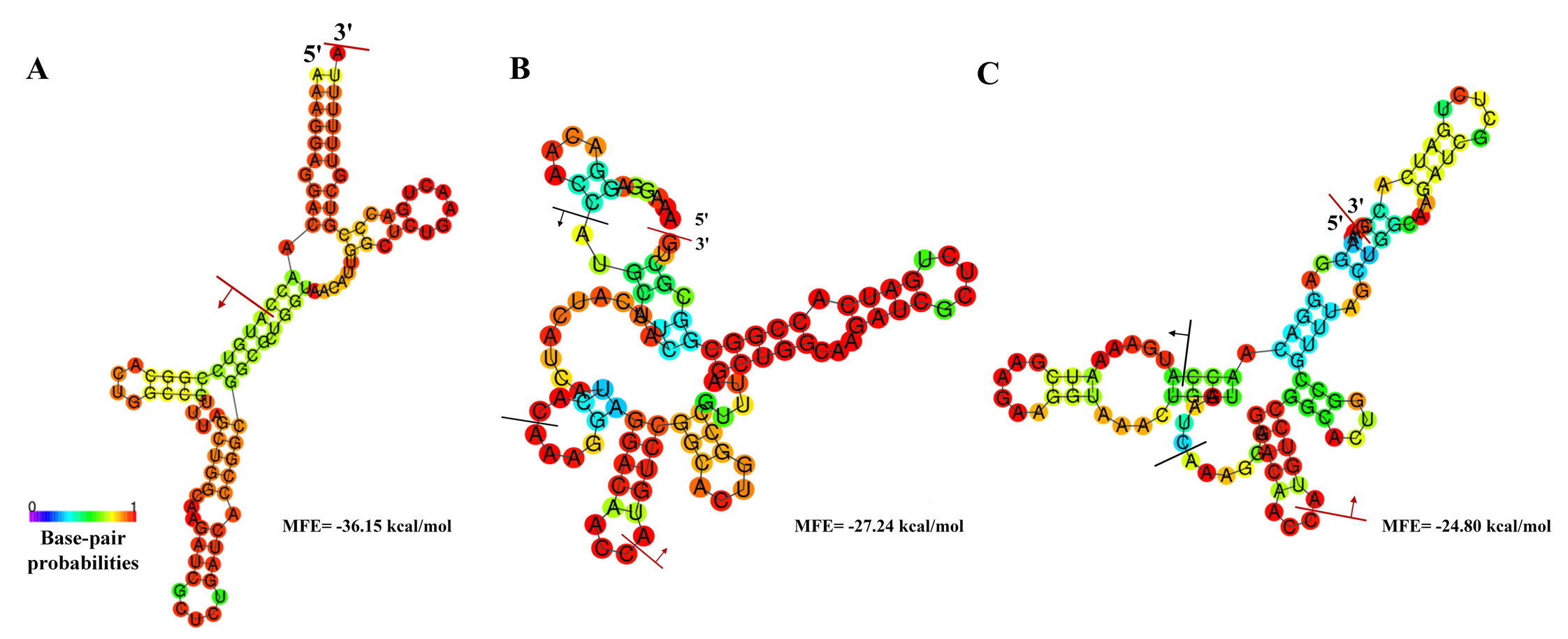


**Supplementary Figure S3.** The mRNA secondary structure of (A) pEC-*mcr**. The mRNA secondary structure of pEC-*mcr** includes RBS sequence and the codons of *mcr* 5’ terminal at a total length of 104 nucleotides. The *mcr* 5’initial sequence starts with the red arrow and ends with the red bar at 3’ end. The minimum free energy (MFE) is –36.15 kcal/mol. (B) pEC-his-*mcr**. The mRNA secondary structure of pEC-his-*mcr** includes RBS sequence, his tag and the codons of *mcr* 5’ terminal at a total length of 104 nucleotides. Tag his sequence starts with the black arrow and ends with the black bar; The *mcr* 5’ initial sequence starts with the red arrow and ends with the red bar at 3’ end. The minimum free energy (MFE) is –27.24 kcal/mol. (C) pEC-mbp-*mcr**. The mRNA secondary structure of pEC-mbp-*mcr** includes RBS sequence, mbp tag and the codons of *mcr* 5’ terminal at a total length of 104 nucleotides. Tag mbp sequence starts with the black arrow and ends with the black bar; The *mcr* 5’ initial sequence starts with the red arrow and ends with the red bar at 3’ end. The minimum free energy (MFE) is –24.8 kcal/mol. The mRNA secondary structures were analyzed by RNAfold Web Server. Only the folding temperature of 30 °C was changed among all constraints.


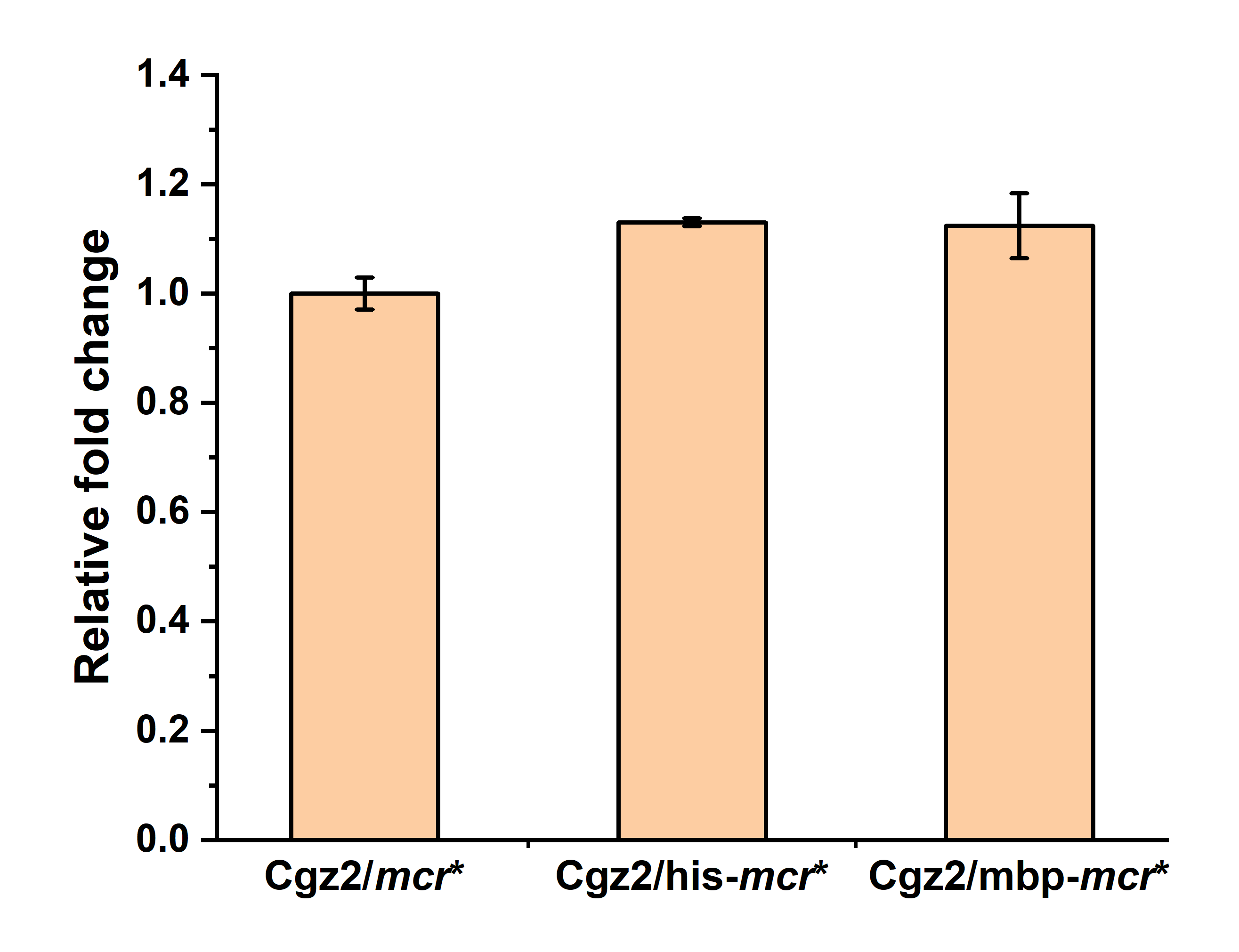


**Supplementary Figure S4.** Relative mRNA levels of gene *mcr** in strains Cgz2/*mcr**, Cgz2/his-*mcr** and Cgz2/mbp-*mcr**. Transcription level of gene *mcr** in Cgz2/*mcr** was considered to be 1.0.


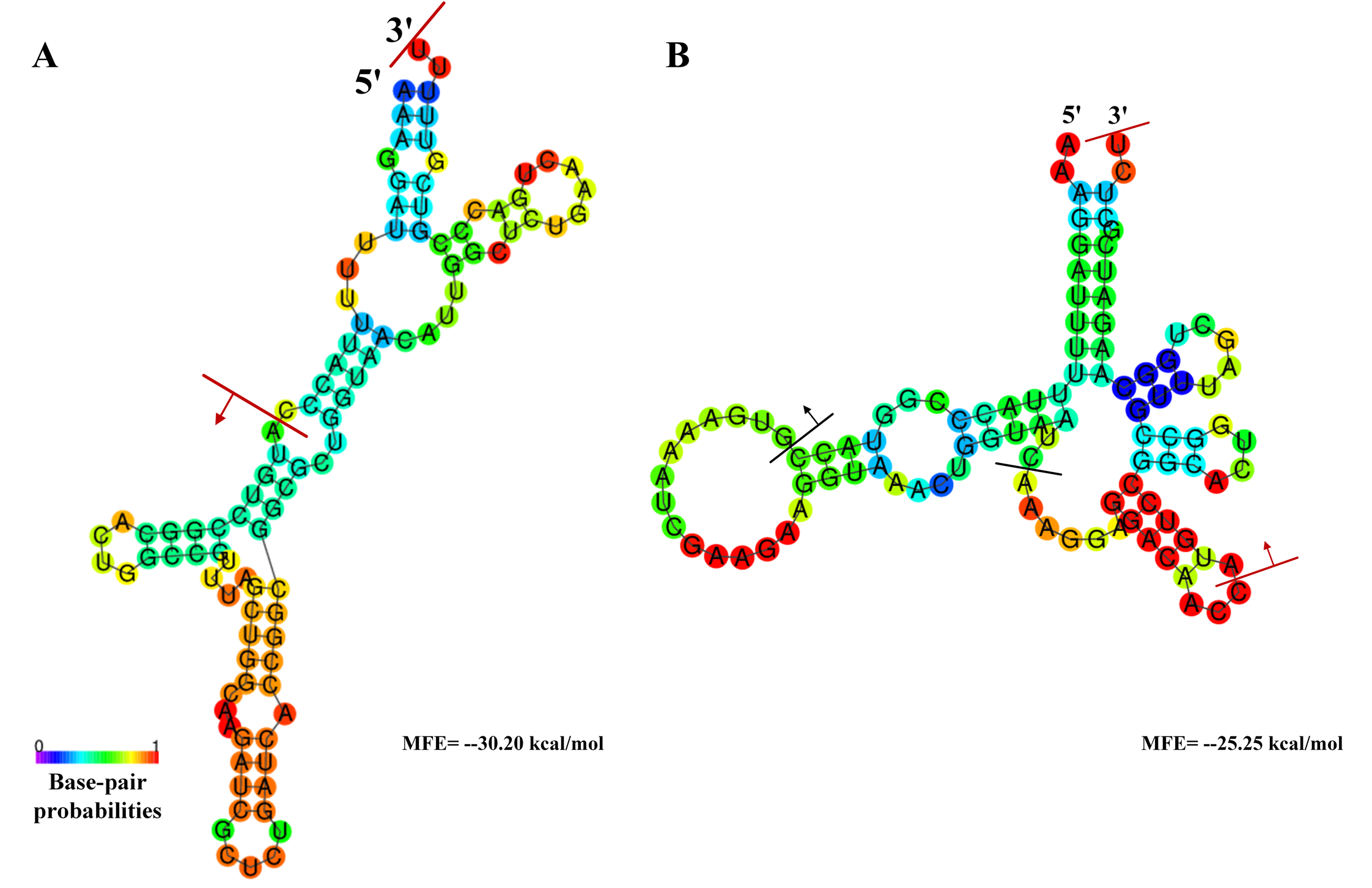


**Supplementary Figure S5.** The mRNA secondary structure of (A) pEC-sod-N-C*. The mRNA secondary structure of pEC-sod-N-C* includes RBS sequence and the codons of *mcr* 5’ terminal at a total length of 104 nucleotides. The *mcr* 5’ initial sequence starts with the red arrow and ends with the red bar at 3’ end. The minimum free energy (MFE) is –30.2 kcal/mol. (B) pEC-sod-mbp-N-C*. The mRNA secondary structure of pEC-sod-mbp-N-C* includes RBS sequence, mbp tag and the codons of *mcr* 5’ terminal at a total length of 104 nucleotides. Tag mbp sequence starts with the black arrow and ends with the black bar; The *mcr* 5’ initial sequence starts with the red arrow and ends with the red bar at 3’ end. The minimum free energy (MFE) is –25.25 kcal/mol. The mRNA secondary structures were analyzed by RNAfold Web Server. Only the folding temperature of 30 °C was changed among all constraints.


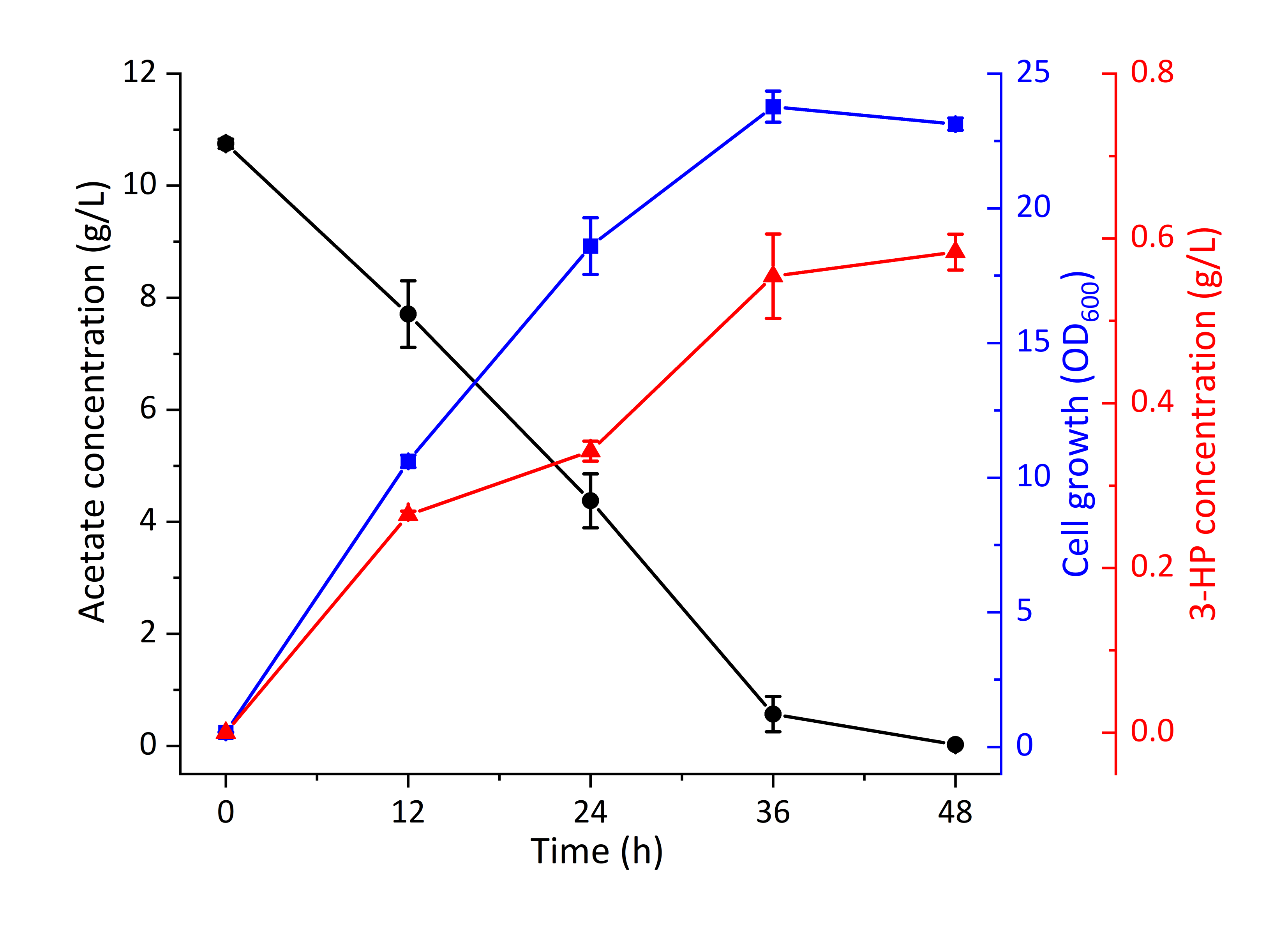


**Supplementary Figure S6.** Time profiles of the cell growth (OD_600_), acetate and 3-HP concentrations of recombinant strain Cgz2/sod-mbp-N-C*.


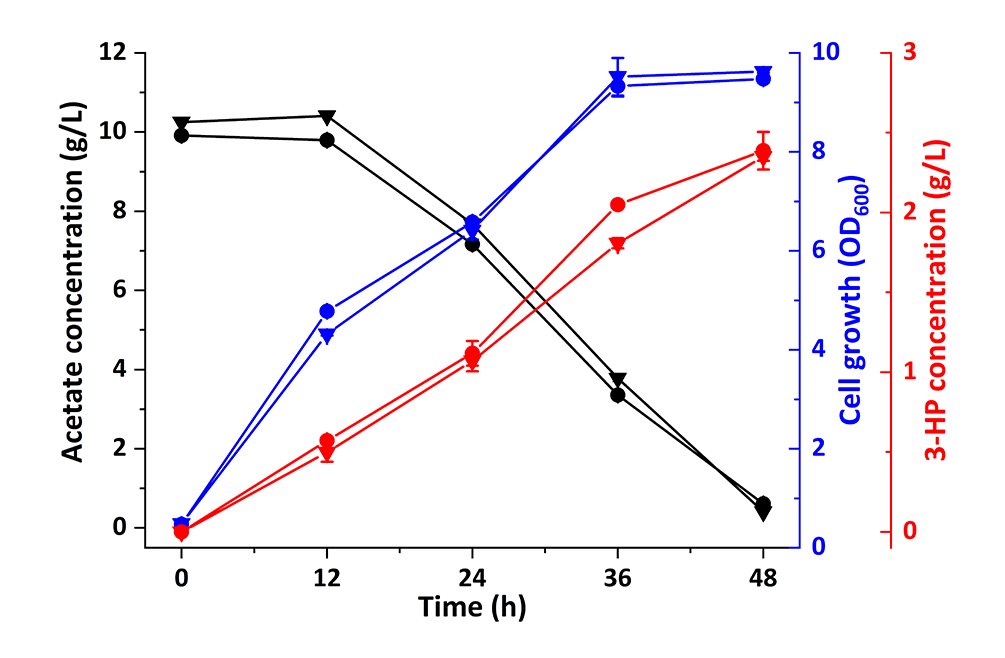


**Supplementary Figure S7.** Time profiles of the cell growth (OD_600_), acetate and 3-HP concentrations of recombinant strains Cgz12/sod-N-C* and Cgz14/sod-N-C*. Circles indicate strain Cgz12/sod-N-C*; Triangles indicate strain Cgz14/sod-N-C*.


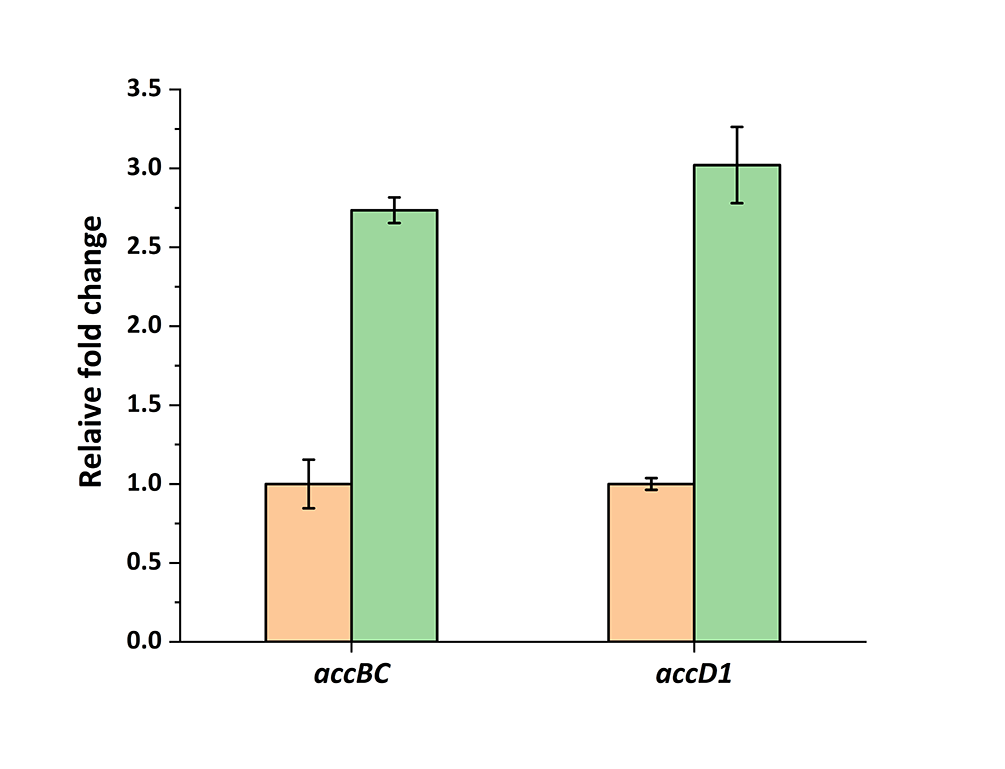


**Supplementary Figure S8.** Relative mRNA levels of genes *accBC* and *accD1* in strains Cgz12/sod-N-C* and Cgz14/sod-N-C*. Orange bars indicate strain Cgz12/sod-N-C*, green bars indicate strain Cgz14/sod-N-C*. Transcription levels of genes *accBC* and *accD1* in Cgz12/sod-N-C* were considered to be 1.0.

## Supplementary Tables

**Supplementary Table S1** Primers used in this study

| Primers | Sequence | Purpose |  |
| --- | --- | --- | --- |
| *mcr*-1 | TTCGGGAGCTCAAAGGAGGACAACCATGTCCGGCACTGGCCGTTTAGCTG | To construct pEC-*mcr* and pEC-*mcr** and pEC-N-C and pEC-N-C* |  |
| *mcr*-2 | AGAGCGAGCGCCGGTGGTTGCAGAAATATTAGCGGGGATGTTTAAAGTG | To construct pEC-*mcr* |  |
| *mcr*-3 | CACTTTAAACATCCCCGCTAATATTTCTGCAACCACCGGCGCTCGCTCT | To construct pEC-*mcr* |  |
| *mcr*-4 | TTCAAGGTACCTTACACAGTGATTGCGCGAC | To construct pEC-*mcr* and pEC-mbp-*mcr** and pEC-his-*mcr** |  |
| *mcr**-1 | GGAACGAGCGCCGGTGGTTGCAGAAATATTAGCGGGGATGTTTAAAG | To construct pEC-*mcr** |  |
| *mcr**-2 | CACTTTAAACATCCCCGCTAATATTTCTGCAACCACCGGCGCTCGTTCC | To construct pEC-*mcr** |  |
| *mcr**-3 | TTCAAGGTACCTTACACGGTGATAGCACGACCAC | To construct pEC-*mcr** |  |
| mbp-*mcr**-1 | CGGAGGAGCTCAAAGGAGGACAACCATGAAAATCGAAGAAGGTAAACTGGTAATCAAAGGAGGACAACCATGTCCGGCACTGGCCGTTTAGCTG | To construct pEC-mbp-*mcr** |  |
| his-*mcr**-1 | CCAAAGAGCTCAAAGGAGGACAACCATGCATCATCATCATCATCACAAAGGAGGACAACCATGTCCGGCACTGGCCGTTTAGCTGGCAAGATCG | To construct pEC-his-*mcr** |  |
| N-C-1 | TTCAAGGTACCTTAAATATTAGCGGGGATGTTTAAAGTGATCTC | To construct pEC-N-C and pEC-N-C* |  |
| N-C-2 | AGCTAGGTACCAAAGGAGGACAACCATGTCTGCAACCACCGGCGCTCGTTC | To construct pEC-N-C and pEC-N-C* and pEC-C*-N |  |
| N-C-3 | AGCTAGGATCCTTACACGGTGATAGCACGACCACGGTGGATG | To construct pEC-N-C and pEC-N-C* and pEC-C*-N |  |
| C*-N-1 | TTCGGGGATCCAAAGGAGGACAACCATGTCCGGCACTGGCCGTTTAGCTG | To construct pEC-C*-N |  |
| C*-N-2 | TTCAATCTAGATTAAATATTAGCGGGGATGTTTAAAGTGATCTC | To construct pEC-C*-N |  |
| H36-N-C*-1 | TTATAGGTACCAAAGGAGGACAACCATGTCCGGCACTGGCCGTTTAGCTG | To construct pEC-H36-N-C* |  |
| H36-N-C*-2 | CGAATTCTAGATTACACGGTGATAGCACGACCAC | To construct pEC-H36-N-C* and pEC-sod-mbp-N-C* |  |
| H36-N-C*-3 | CGTCATCTAGAGTCGACCTGCAGGCATGCAAG | To construct pEC-H36-N-C* and pEC-sod-mbp-N-C* |  |
| H36-N-C*-4 | CTCCAGGTACCCATGCTACTCCTACCAAC | To construct pEC-H36-N-C* |  |
| sod-N-C*-1 | TTCAACTTAAGGAATTCTAGCTGCCAATTATTCCGGGCTTGT | To construct pEC-sod-N-C* |  |
| sod-N-C*-2 | CAGCTAAACGGCCAGTGCCGGACATGGGTAAAAAATCCTTTCGTAGGTT | To construct pEC-sod-N-C* |  |
| sod-N-C*-3 | AACCTACGAAAGGATTTTTTACCCATGTCCGGCACTGGCCGTTTAGCTG | To construct pEC-sod-N-C* |  |
| sod-N-C*-4 | TCCGTGAATTCCTTAAGATTCACCACCCTGAATTGACTCTCTTCC | To construct pEC-sod-N-C* |  |
| sod-mbp-N-C*-1 | AATCTGGTACCGTGAAAATCGAAGAAGGTAAACTGGTAATCAAAGGAGGACAACCATGTCCGGCACTGGCCGTTTAGCTG | To construct pEC-sod-mbp-N-C* |  |
| sod-mbp-N-C*-2 | CGCATGGTACCGGGTAAAAAATCCTTTCGT | To construct pEC-sod-mbp-N-C* |  |
| *gltA*-1 | TTAAAGAATTCTACTGGCAGTCCACCAGCGCCGGAG | To construct pD-*sacB*-P1-*gltA* and pD-*sacB*-P5-*gltA* and pD-*sacB*-P7-*gltA* |  |
| *gltA*-2 | ATGGATCTAGAATGTTGGCCTGTGCGGAACCGAT | To construct pD-*sacB*-P1-*gltA* and pD-*sacB*-P5-*gltA* and pD-*sacB*-P7-*gltA* |  |
| *gltA*-3 | AGTTTTGCAAAGTTTTCAATTTCAAAATTATTTTAAATTTGTGCTTGA | To construct pD-*sacB*-P1-*gltA* |  |
| *gltA*-4 | TCAAGCACAAATTTAAAATAATTTTGAAATTGAAAACTTTGCAAAACT | To construct pD-*sacB*-P1-*gltA* |  |
| *gltA*-5 | GCATTGTGGTATAATGGACCAGTGCAAAGGAGGACAACCATGTTTGAAAGGGATATCGT | To construct pD-*sacB*-P1-*gltA* |  |
| *gltA*-6 | ACGATATCCCTTTCAAACATGGTTGTCCTCCTTTGCACTGGTCCATTATACCACAATGC | To construct pD-*sacB*-P1-*gltA* |  |
| *gltA*-7 | AGTTTTGCAAAGTTTTCAATTTCAAAATTATTTTAAATTTAGTATTGA | To construct pD-*sacB*-P5-*gltA* |  |
| *gltA*-8 | TCAATACTAAATTTAAAATAATTTTGAAATTGAAAACTTTGCAAAACT | To construct pD-*sacB*-P5-*gltA* |  |
| *gltA*-9 | CATCTGTGATACAATGGGATAGTGCAAAGGAGGACAACCATGTTTGAAAGGGATATCGT | To construct pD-*sacB*-P5-*gltA* |  |
| *gltA*-10 | ACGATATCCCTTTCAAACATGGTTGTCCTCCTTTGCACTATCCCATTGTATCACAGATG | To construct pD-*sacB*-P5-*gltA* |  |
| *gltA*-11 | AGTTTTGCAAAGTTTTCAATTTCAAAATTATTTTAAATTTCACATTGA | To construct pD-*sacB*-P7-*gltA* |  |
| *gltA*-12 | TCAATGTGAAATTTAAAATAATTTTGAAATTGAAAACTTTGCAAAACT | To construct pD-*sacB*-P7-*gltA* |  |
| *gltA*-13 | CATTGTGATACAATGGTAGAGTGCAAAGGAGGACAACCATGTTTGAAAGGGATATCGT | To construct pD-*sacB*-P7-*gltA* |  |
| *gltA*-14 | ACGATATCCCTTTCAAACATGGTTGTCCTCCTTTGCACTCTACCATTGTATCACAATG | To construct pD-*sacB*-P7-*gltA* |  |
| *gltA*-15 | GGAGGACAACCGTGTTTGAAAGGGA | To construct pD-*sacB*-P1-GTG-*gltA* |  |
| *gltA*-16 | TCCCTTTCAAACACGGTTGTCCTCC | To construct pD-*sacB*-P1-GTG-*gltA* |  |
| *gltA*-17 | GGAGGACAACCTTGTTTGAAAGGGA | To construct pD-*sacB*-P1-TTG-*gltA* |  |
| *gltA*-18 | TCCCTTTCAAACAAGGTTGTCCTCC | To construct pD-*sacB*-P1-TTG-*gltA* |  |
| *accBC*-1 | CCTTAGGATCCAATGAAGATTCCCTT | To construct pD-*sacB*-*fasO*(M)-*accBC* |  |
| *accBC*-2 | TGCTTACTTACGACTATTCTGGGGGAATTCTTCTGTTTTAGGC | To construct pD-*sacB*-*fasO*(M)-*accBC* |  |
| *accBC*-3 | CCCCAGAATAGTCGTAAGTAAGCATATCTGGTTGAGTTCTTCGGGGTT | To construct pD-*sacB*-*fasO*(M)-*accBC* |  |
| *accBC*-4 | CGATCTCTAGACGAGGTACTCAACGGTGC | To construct pD-*sacB*-*fasO*(M)-*accBC* |  |
| *accD1*-1 | TTCACGAATTCTCGCGAACACGAATTTCTCC | To construct pD-*sacB*-*fasO*(M)-*accD1* |  |
| *accD1*-2 | AAGGGCTACTAATGGTCATGTTTTGAAATC | To construct pD-*sacB*-*fasO*(M)-*accD1* |  |
| *accD1*-3 | AAACATGACCATTAGTAGCCCTTTGATTGACGTCGCCAAC | To construct pD-*sacB*-*fasO*(M)-*accD1* |  |
| *accD1*-4 | TCACTTCTAGACTTCAACGCCGCCTTCTTCCTCGTC | To construct pD-*sacB*-*fasO*(M)-*accD1* |  |

The underlined sequences correspond to restriction enzyme sites.
